# Impact of definition of meibomian gland dysfunction on the frequency of MGD in dry eye disease from the DREAM© study

**DOI:** 10.1016/j.clae.2025.102416

**Published:** 2025-04-12

**Authors:** Divya Jagadeesh, Meng C. Lin, Fiona Stapleton, Jocelyn He, Gui-Shuang Ying, Penny Asbell

**Affiliations:** a School of Optometry and Vision Science, UNSW Sydney, Australia; b Clinical Research Center, Herbert Wertheim School of Optometry and Vision Science, University of California, Berkeley, USA; c Department of Ophthalmology, Scheie Eye Institute, University of Pennsylvania, Philadelphia, PA, USA; d Bioengineering Department, University of Memphis, Tennessee, and School of Optometry and Vision Science, UNSW Sydney, Australia

**Keywords:** Meibomian gland dysfunction, Dry eye disease, Plugging, Secretion, Atrophy, Demographic factors

## Abstract

**Purpose:**

Meibomian gland dysfunction (MGD) is a common disease, yet there is no standard clinical definition. This study explored the frequency and characteristics of MGD in dry eye disease (DED) using different clinical definitions.

**Methods:**

A secondary analysis of data collected from the DREAM© study participants (n = 535) with moderate-to-severe DED was undertaken. Three common MG clinical features; plugging, secretion quality, and MG atrophy grade, either separately or in combination were used to create MGD definitions. The frequency of MGD, defined by the per eye findings, was reported. Demographic characteristics were compared between those with and without MGD.

**Results:**

The frequency of MGD in DED varied from 12 % (gland plugging, plus poor secretion quality plus gland atrophy) to 55 % (gland plugging alone) of eyes. Older age was associated with MGD when atrophy-based definitions were used (*p* < 0.001) but was not associated with MGD definitions using plugging or secretion quality (*p* > 0.05). After adjustment for age and between-eye correlations, MGD frequency was higher in women for definitions based on lower lid gland atrophy (adjusted *p* < 0.05). Hispanic or Latino ethnicity was more commonly associated with MGD than other ethnicities (adjusted *p* < 0.001), for definitions based on plugging and secretion quality.

**Conclusion:**

The frequency of MGD in DED varied with MGD definition. The highest frequency occurred where MGD was defined as lower eyelid gland plugging. Gland atrophy increased with age. The variation in disease frequency has implications for clinical diagnosis and determining efficacy assessment in intervention studies.

## 1. Introduction

Meibomian gland dysfunction (MGD) is a common condition that disrupts ocular surface homeostasis, yet there is no standard clinical definition for MGD for use in epidemiological and clinical studies or in a clinical setting. MGD is defined by the Tear Film and Ocular Surface Society (TFOS) MGD workshop as “*a chronic diffuse abnormality of the meibomian glands commonly characterized by terminal duct obstruction and qualitative and/or quantitative changes in glandular secretion. It may result in alteration of the tear film, symptoms of eye irritation, clinically apparent inflammation, and ocular surface disease”* [1]. Valid endpoints need to be described to operationalise this definition [2] to better establish prevalence and explore associations and risk factors but the impact of different clinical definitions is unknown. In addition, MGD is hypothesized to be a key contributor to dry eye disease (DED) [3] from epidemiological studies, but there are limited data on well characterized DED cohorts which describe the prevalence of MGD in DED. The recommendation from the TFOS Society consensus workshop is to consider (a) morphologic lid features, (b) gland expressibility, and (c) gland dropout using meibography for the diagnosis and characterization of MGD [1]. While MGD is frequently associated with DED, it is also recognised that a significant proportion of the disease is asymptomatic [4], (Stage 1 MGD) and that this proportion may change with age [5].

The population prevalence of MGD ranges widely from 21 % to 71 % based on a *meta*-analysis comprising studies using different disease definitions, conducted in different geographic regions with a pooled prevalence of 35.8 % (95 % confidence intervals 22–52) [6]. Existing diagnostic criteria use either a single or composite score and use features such as the number of patent or obstructed glands, characteristics of eyelid margins e.g., thickness, rounding, vascularity, staining, location of Marx line, as well as gland dropout, gland area, shape, orifices, plugging, meibum expressibility, quality and/or volume [7–12].

The Dry Eye Assessment and Management Study (DREAM©) was a randomized controlled trial where investigators at 27 clinical centres in the United States evaluated the safety and efficacy of ω−3 fatty acid supplementation to improve dry eye symptoms based on the Ocular Surface Disease Index (OSDI) score [13]. Diagnostic tests for MGD were preferentially selected as those commonly performed in clinical practice.

The aim of this study is to undertake a secondary analysis of baseline (pre-intervention) characteristics in participants from the DREAM© study to explore (a) the frequency of MGD with DED, using diagnostic criteria for MGD based on clinical observations of structural and functional changes to the glands (b) the impact of MGD definition on associations between MGD and demographic factors, namely age, sex and ethnic background.

## 2. Methods

The DREAM© study was approved by the institutional review board of all the respective 27 examination centres (Clinicaltrials.gov NCT02128763) and adhered to the tenets of the Declaration of Helsinki. The design and the baseline characteristics of the study have been previously published [13]. Informed consent was obtained before data collection.

This double-masked, placebo-controlled randomized controlled, safety and efficacy trial studied ω−3 fatty acid supplementation in subjects with moderate-to-severe DED. Inclusion criteria, in brief, were: age ≥18 years; at least 2 of 4 ocular signs present at the baseline visit – Lissamine green conjunctival staining ≥1 (0–6 per modified NEI scale [13]), corneal fluorescein staining ≥4 (0–15 per NEI scale [13], tear film break-up time ≤7 s and anesthetized Schirmer’s test ≥1 to ≤7 mm/5 min; and symptoms of DED (OSDI score of at least 25), patient-reported dry eye symptoms and use or desire to use artificial tears and intraocular pressure ≥5 mmHg and ≤22 mmHg. Exclusion criteria included contact lens wear, pregnancy, or a history of previous ocular surgery or refractive surgery [13]. Clinical signs were evaluated after symptoms and in the following order; slit lamp evaluation, tear break up time, corneal fluorescein staining, meibomian gland examination, lissamine green staining and Schirmer test [13].

### 2.1. Assessment of ocular symptoms and signs

Participants completed a dry eye treatment history form and OSDI questionnaire. The assessment of signs of DED has been previously reported [13,14]. MGD was evaluated in each eye. The meibomian gland evaluator (MGE, Tear Science, Morrisville, NC) was used for the evaluation of MG plugging and quality of meibum of the lower eyelid. Plugging and secretion quality were reported for the lower eyelid only. The lower eyelid margin glands are more easily accessible and visible than those in the upper lid and the lower eyelid arguably is less susceptible to reflex blinking than the upper eyelid. Gland plugging was graded from 0 to 3, based on the number of glands plugged, and the meibum quality was also graded from 0 to 3 (Table 1) [13]. Meibomian gland loss or dropout in the upper and lower eyelids was assessed using meibography images (available for 292 patients) obtained at the centres where the Oculus Keratograph 5M was available (10/27 centres) [15]. Upper and lower eyelid MG images were captured to assess whether atrophy levels differed between the upper and lower eyelid. MG atrophy was graded by a single masked observer and the extent of gland loss was graded using the Pult 5-Grade scale [16] (Table 1). At the ten centres where the Oculus Keratograph 5M was available, meibography was conducted prior to the slit lamp evaluation [13]. Meibography was not conducted at the other 17 centres.

These parameters were initially selected due to their accessibility and acceptability in clinical practice.

### 2.2. MGD definitions

Based on the TFOS recommendations and considering commonly described clinical features, the following criteria were applied to develop definitions for MGD.

1. MG plugging ≥2 (range 0–3) [7],
2. Quality of the gland secretion ≥2 (range 0–3) [1],
3. MG loss or dropout based on a Pult score ≥2 (range 0–4) for the upper eyelid [16],
4. MG loss or dropout based on Pult score ≥2 (range 0–4) for the lower eyelid [16].

These criteria were used for each eye either separately or in combination, that is where there is more than one sign in the same eye, to create 9 diagnostic criteria for MGD (Tables 1 and 2).

### 2.3. Statistical analysis

The frequency of MGD for each of the nine diagnostic criteria was estimated by eye that is, each eye was included individually in the analysis, and frequency was reported as the percentage of eyes affected. As previous publications established an effect of age on MGD [17,18], initial analysis explored the effect of age on MGD frequency for each diagnostic criteria while adjusting for inter-eye correlations using generalized linear regression analysis. Generalized linear regression models, with Wald test, were subsequently applied for each definition to assess the effects of sex and ethnicity on MGD frequency, after adjusting for age and inter-eye correlations. Bonferroni correction was applied to adjust for multiple comparisons across the nine MGD definitions. Associations between categorical grades of plugging, secretion quality and atrophy were evaluated using the Spearman rank correlation coefficient (r). Adjusted p values are reported, statistical significance was set at 5 % and analysis was conducted using SPSS (Version 28.0, Armonk, NY).

## 3. Results

Data for gland plugging and secretion were available for 535 participants and images for MG atrophy for 292 participants. The mean age of the study population was 58 ± 13 and 81 % were women. The frequency estimates of MGD in those with moderate-to-severe DED varied from 12 to 55 % depending on the diagnostic criteria used (Table 3). Lower lid gland plugging ≥grade 2 (definition 1) was observed in 55 % of the sample, followed by atrophy of the glands of the lower lid (definition 4) in 45 %. The least common was when the disease was diagnosed through a combination (all signs present) of plugging and secretion of the lower lids, plus atrophy of the lower and upper lid ≥grade 2 (definition 9) in 12 %.

For all atrophy-based definitions, age was significantly higher in those with MGD compared to those without MGD (*p* < 0.001, Table 4). However, age was not significantly associated with plugging or secretion-based definitions of MGD (Table 4).

Table 5 summarizes associations between MGD definitions and participant sex, and ethnicity. The frequency of MGD was higher in women for diagnoses including atrophy of the lower lid (definitions 4 and 8; *p* < 0.05). There was no significant difference between males and females in the frequency of MGD for diagnoses based on plugging or secretion quality, after adjusting for age and inter-eye correlations.

For ethnic background, Hispanic or Latino participants comprised 12.7 % of the sample, White (69.2 %), Black (12.0 %), and other/unknown (6.2 %). Compared with Black or White, Hispanic or Latino ethnic backgrounds were associated with a higher frequency of MGD based on plugging or secretion quality or composite criteria including those parameters (i.e., definitions (1, 2, 7, and 8, Table 5), after adjusting for age and inter-eye correlations.

There was a strong association between lower lid plugging and secretion scores (*r* = 0.72, *p* < 0.001), but a much weaker association between atrophy and plugging (*r* = 0.11, *p* < 0.05). Other associations between scales did not reach statistical significance.

## 4. Discussion

This study has explored the frequency of MGD using a range of diagnostic criteria based on characteristics of meibomian glands commonly evaluated in clinical practice, namely gland plugging, meibum secretion quality, and upper and lower lid gland atrophy. It also explored the effects of demographic characteristics (age, sex, and ethnic background) on disease characteristics in MGD [19].

In a population with moderate-to-severe DED, MGD frequency varied with diagnostic criteria, consistent with previous reports of population prevalence of MGD [6] and in clinic-based studies of patients with DED [20]. Twelve percent of eyes with DED in this study showed all three signs of MGD evidenced by gland plugging, plus altered secretion quality plus gland atrophy. The lower eyelid gland plugging was a common characteristic, observed in 55 % of eyes with moderate-to-severe DED. Altered secretion quality occurred in 41 %. Significant gland atrophy was observed in 43 % of the upper and 45 % of the lower lids, which is consistent with earlier work showing a similar rate of atrophy between the eyelids [10,21–23]. This variation in disease frequency with different criteria underscores the importance of disease-definition standardization which is critical for tracking longitudinal changes and understanding the natural history of the disease and treatment response [24].

Previous studies have suggested that demographic characteristics influence disease presentation and severity [25], with increasing age and South-East Asian ethnicity associated with increased risk of disease. Consequently, preliminary analysis explored the effect of age on rates of disease based on the different diagnostic criteria. In the DREAM© cohort of moderate-to-severe DED, age was not associated with MGD defined by gland plugging and secretion quality, but was significantly associated with MGD defined by atrophy, suggesting that gland atrophy is age-related. The age-related changes in MG have been reported previously [26,27]. The strong association between lower lid plugging and secretion scores, and considerably weaker association between atrophy and plugging is consistent with a previous report [28]. This may suggest that plugging and secretion quality are linked and perhaps occur independently of the development of atrophy, but this requires further exploration, including elucidation of the natural history of these features [29,30].

This age association with atrophy but not with other features is perhaps unexpected given the established age-related change in meibum composition [31]. Evidence for the effects of age on meibomian glands, however, has predominantly arisen from animal studies that have used gland atrophy as a surrogate for MGD [32,33], given it is difficult to evaluate gland obstruction and meibum in animal eyelids. Extensive gland atrophy, however, in animal models may better correlate with severe MGD in humans [34]. The observations that atrophy is related to age and the fact that short glands can be functional (i.e., expressing clear meibum) would suggest atrophy is not a good marker for MGD in humans [30].

The effect of sex on MGD is equivocal and may vary with diagnostic criteria [3,13,35]. When controlling for age and the effect of inter-eye correlations, women had a higher rate of disease diagnosed using lower eyelid gland atrophy, either alone or in combination with other parameters. Although this finding is consistent with results from a population-based cohort showing a higher rate of atrophy in women [36], caution must be exercised in the interpretation as the rate of lower lid atrophy may be difficult to estimate due to the difficulty in consistently defining the tarsal plate area of the lower lid [37,38]. This lack of a robust association between sex and altered secretion or gland plugging is perhaps surprising given the strong association between women and DED [3] and that the majority of DED is often attributed to MGD [39,40].

In exploring the relationship between ethnicity and the definition of MGD, this study did not find a higher rate of MGD in those with an Asian ethnic background likely due to the small sample size of Asian participants. A previous study reported greater meibomian gland density and fewer ghost glands in Asian ethnicity when compared to Caucasians [41]. In this cohort, Hispanic or Latino ethnicity was associated with a higher rate of disease defined using gland plugging and secretion quality. This has previously been reported in both MGD [6] and DED [42]. Compared with White individuals, altered meibum secretion, namely a lower cholesterol ester to wax ester ratio, consistent with that seen in MGD, has been shown in Hispanics, although the sample size in this study was small [31].

Strengths of the study include many clinical centres in diverse geographical locations in the United States, the inclusion of a large well-characterised DED group with moderate-to-severe disease and the use of accessible and commonly used clinical techniques to define MGD. Limitations include that atrophy data were available from ten of the 27 centres, who had access to meibography imaging, consequently the smaller sample size may limit the generalisability of the atrophy data. Additionally, gland plugging and meibum expression were performed only on lower lids. No quantitative measure of lipid production was included, as this was not part of the original DREAM© study protocol. Clinical parameters in line with the definition of MGD that are accessible in routine clinical practice were preferentially selected. Finally, participants had moderate-to-severe DED, such that the findings may not be generalisable to those with mild disease or to a contact lens-wearing population with MGD.

These findings can be used to inform the natural history of MGD in treated and untreated disease and may also suggest that secretion quality and gland plugging may be more appropriate measures of MGD rather than atrophy which varies with age and sex. Secretion quality, for example, is responsive to treatment for MGD [43]. Importantly, a prior history of MGD (based on medical records) has been identified as a predictor for the future development of severe DED [44] and the time course of development of signs may guide the timing, duration, and cessation of intervention.

## 5. Conclusions

This study has confirmed a variation in the frequency of MGD in DED based on diagnostic criteria. This variation in disease frequency has implications for understanding clinical diagnosis of MGD, the natural history of MGD and for efficacy evaluation in intervention studies. Upper and lower eyelid atrophy vary with both age and sex, however, diagnoses based on gland plugging and secretion quality are less affected by these demographic variables although Hispanic ethnicity is associated with a higher rate of MGD based on these criteria.

## Figures and Tables

**Table 1:** Grading of plugging, quality of meibum and MG loss or dropout.

Plugging^a^		Quality of meibum (secretion)^a^		MG atrophy (Pult grade)^b^	
Grade (0 to 3)	No. of glands plugged	Grade (0 to 3)	Feature	Grade (0 to 4)	Gland loss
(0)	None	(0)	Clear	(0)	0 %
(1)	1 to 2	(1)	Mild haze/cloudiness	(1)	<25 %
(2)	3 to 4	(2)	Paste (like toothpaste)	(2)	25 %–50 %
(3)	5	(3)	Obstructed (no secretions)	(3)	51 %–75 %
				(4)	>75 %

a Asbell et al., 2018 [13] Central 5 glands assessed;

b Pult et al. 2016 [16]; Meibography images available in 292 participants.

**Table 2:** Diagnostic criteria.

No:	Criteria for MGD	Short definition
1	Plugging (lower lid only): MGD = ≥ grade 2	Plugging LL
2	Quality of meibum (lower lid only): MGD = ≥ grade 2	Secretion LL
3	Pult MG atrophy (upper lid): MGD = ≥ grade 2	Atrophy UL
4	Pult MG atrophy (lower lid) MGD = ≥ grade 2	Atrophy LL
5	(Definition 1 + definition 2)	Plugging LL + Secretion LL
6	(Definition 3 + definition 4)	Atrophy UL + LL
7	(Definition 1 + definition 2 + definition 3)	Plugging LL + Secretion LL + Atrophy UL
8	(Definition 1 + definition 2 + definition 4)	Plugging LL + Secretion LL + Atrophy LL
9	(Definition 1 + definition 2 + definition 3 + definition 4)	All combined

*LL* – *lower lid; UL* – *Upper lid*.

**Table 3:** Frequency of MGD.

No:	Short definition	Eyes with MGD (frequency)	Total (eyes)
1	Plugging LL	589 (55 %)	1070
2	Secretion LL	438 (41 %)	1070
3	Atrophy UL	218 (43 %)	502
4	Atrophy LL	247 (45 %)	555
5	Plugging LL + Secretion LL	362 (34 %)	1070
6	Atrophy UL + LL	144 (29 %)	499
7	Plugging LL + Secretion LL + Atrophy UL	102 (20 %)	502
8	Plugging LL + Secretion LL + Atrophy LL	117 (21 %)	555
9	All combined	61 (12 %)	499

LL – lower lid; UL – Upper lid.

**Table 4:** Age in those with and without MGD.

No:	MGD definition	Age (years), Mean ± SD		
		With MGD	Without MGD	Inter-eye adjusted p-value
1	Plugging LL	58.6 ± 12.6	57.3 ± 13.9	0.10
2	Secretion LL	58.4 ± 12.5	57.7 ±13.7	0.39
3	Atrophy UL	60.6 ± 12.8	53.5 ± 14.5	<0.001
4	Atrophy LL	60.5 ± 11.4	53.1 ± 14.9	<0.001
5	Plugging LL + Secretion LL	58.5 ± 12.6	57.8 ± 13.5	0.39
6	Atrophy UL + LL	62.0 ± 12.0	54.3 ± 14.4	<0.001
7	Plugging LL + Secretion LL + Atrophy UL	62.5 ± 12.2	55.1 ± 14.3	<0.001
8	Plugging LL + Secretion LL + Atrophy LL	60.7 ± 10.8	55.3 ± 14.4	<0.001
9	All combined (1 + 2 + 3 + 4)	63.2 ± 11.9	55.6 ± 14.3	<0.001

**Table 5:** Associations between MGD definition and sex and ethnicity.

Study variables (data for both eyes)	Sex			Race				
	Women (n = 434	Men (n = 101)	Age- & inter-eye-adjusted p-value^a^	Hispanic or Latino (n = 68)	White (n = 370)	Black (n = 64)	Other or unknown (n = 33)	Age- & inter-eye-adjusted p-value^b^
	% eyes with MGD	% eyes with MGD		% eyes with MGD	% eyes with MGD	% eyes with MGD	% eyes with MGD	
1. Plugging LL (1070 eyes of 535 patients)	56.0 %	51.0 %	0.23	64.7 %	54.2 %	44.5 %	65.2 %	<0.001
2. Secretion LL (1070 eyes of 535 patients)	42.0 %	36.0 %	0.13	60.0 %	35.0 %	45.0 %	55.0 %	<0.001
3. Atrophy UL (502 eyes of 251 patients)	44.2 %	40.5 %	0.95	32.9 %	46.2 %	34.1 %	48.6 %	0.21
4.Atrophy LL (555 eyes of 288 patients)	47.6 %	33.9 %	0.024	42.3 %	46.3 %	40.4 %	39.0 %	0.87
5. Plugging LL + Secretion LL (n = 535 eyes of 1070 patients)	35.1 %	28.2 %	0.07	50.0 %	30.5 %	28.9 %	47.0 %	<0.001
6.Atrophy UL + LL (n = 499 eyes of 250 patients)	30.7 %	22.5 %	0.027	18.8 %	31.7 %	20.5 %	30.6 %	0.26
7. Plugging LL + Secretion LL + Atrophy UL (502 eyes of 251 patients)	22.0 %	14.4 %	0.21	22.9 %	19.7 %	9.1 %	35.1 %	0.004
8. Plugging LL + Secretion LL + Atrophy LL (555 eyes of 288 patients)	23.7 %	12.1 %	0.012	25.2 %	18.5 %	23.4 %	29.3 %	0.04
9. All combined (n = 499 eyes of 250 patients)	13.7 %	7.2 %	0.15	13.0 %	11.7 %	6.8 %	22.2 %	0.06

a Adjusted p-value reports comparison of differences in frequency of MGD between women and men, based on general linear models with Wald test.

b Adjusted p-value reports comparison of differences in frequency of MGD between different ethnic backgrounds, based on based on general linear models with Wald test.

## Appendix. The members of the DRy Eye Assessment and Management (DREAM) Research Group

Credit Roster for the DRy Eye Assessment And Management (DREAM) Study

**Certified Roles at Clinical Centres**: Clinician (CL); Clinic Coordinator (CC), Data Entry

Staff (DE) Principal Investigator (PI), Technician (T).

**Milton M. Hom (Azusa, CA)**: Milton M. Hom, OD FAAO (PI); Melissa Quintana (CC/T);

Angela Zermeno (CC/T).

**Pendleton Eye Center (Oceanside, CA)**: Robert Pendleton, MD, PhD. (PI); Debra

McCluskey (CC); Diana Amador (T); Ivette Corona (CC/T); Victor Wechter, MD (CL).

**University of California School of Optometry, Berkeley (Berkeley, CA)**: Meng C. Lin,

OD PhD FAAO (PI); Carly Childs (CC); Uyen Do (CC); Mariel Lerma (CC); Wing Li, OD

(T); Zakia Young (CC); Tiffany Yuen, OD (CC/T).

**Clayton Eye Center (Morrow, GA)**: Harvey Dubiner, MD (PI); Heather Ambrosia, OD

(C); Mary Bowser (CC/T); Peter Chen, OD (CL); Helen Dubiner, PharmD, CCRC (CC/T);

Cory Fuller (CC/T); Kristen New (DE); Tu Vy Nguyen (C); Ethen Seville (CC/T); Daniel

Strait, OD (CL); Christopher Wang (CC/T); Stephen Williams (CC/T); Ron Weber, MD

(CL)

**University of Kansas (Prairie Village, KS)** John Sutphin, MD (PI); Miranda Bishara, MD

(CL); Anna Bryan (CC); Asher Ertel (CC/T); Kristie Green (T); Gloria Pantoja, Ashley

Small (CC); Casey Williamson (T).

**Clinical Eye Research of Boston (Boston, MA)**: Jack Greiner, MS, OD, DO, PhD (PI);

EveMarie DiPronio (CC/T); Michael Lindsay (CC/T); Andrew McPherson (CC/T); Paula

Oliver (CC/T); Rina Wu (T).

**Mass Eye & Ear Infirmary (Boston, MA)**: Reza Dana, MD (PI); Tulio Abud (T): Lauren

Adams (T); Marissa Arnofsky (T); Jillian Candlish, COA (T); Pranita Chilakamarri (DE);

Joseph Ciolino, MD (CL); Naomi Crandall (T); Antonio Di Zazzo (T); Merle Fernandes (T);

Mansab Jafri (T); Britta Johnson (T); Ahmed Kheirkhah (T); Sally Kiebdaj (CC/T); Andrew

Mullins (CC/T); Milka Nova (T); Vannarut Satitpitakul (T); Chunyi Shao (T); Kunal Suri

(T); Vijeeta Tadla (CC); Saboo Ujwala (T); Jia Yin MD, PhD (T); Man Yu (T).

**Kellogg Eye Center, University of Michigan (Ann Arbor, MI)**: Roni Shtein, MD (PI);

Christopher Hood, MD (CL); Munira Hussain, MS, COA, CCRP (CC/T); Erin Manno, COT

(T); Laura Rozek, COT (T/DE).

**Minnesota Eye Consultants (Bloomington, MN)**: David R. Hardten, MD FACS (PI);

Kimberly Baker (T); Alex Belsaas (T); Erich Berg (CC/T); Alyson Blakstad, OD (CL); Ken

DauSchmidt (T); Lindsey Fallenstein (CC/T); Ahmad M. Fahmy OD (CL); Mona M. Fahmy

OD FAAO (CL); Ginny Georges (T); Deanna E. Harter (CL); Scott G. Hauswirth, OD (CL);

Madalyn Johnson (T); Ella Meshalkin (T); Rylee Pelzer (CC/T); Joshua Tisdale (CC/T);

JulieAnn C. Wick (CL).

**Tauber Eye Center (Kansas City, MO)**: Joseph Tauber, MD, PHD (PI); Megan Hefter

(CC/T)

**Silverstein Eye Centers (Kansas City, MO)**: Steven Silverstein, MD (PI); Cindy Bentley

(CC/T); Eddie Dominguez (CC/T); Kelsey Kleinsasser, OD (CL).

Icahn School of Medicine at Mt. Sinai, (New York, NY): Penny Asbell, MD, FACS, MBA

(PI); Brendan Barry (CC/T); Eric Kuklinski (CC/T); Afsana Amir (CC/T); Neil Chen

(CC/T); Marko Oydanich (CC/T); Viola Spahiu (CC/T); An Vo, MD (T); Matthew

Weinstein, DO (T).

**University of Rochester Flaum Eye Institute (Rochester, NY)**: Tara Vaz, OD (PI); Holly

Hindman, MD (PI); Rachel Aleese (CC/T); Andrea Czubinski (CC/T); Gary Gagarinas,

COMT CCRA (CC/T); Peter McDowell (CC); George O’Gara (DE); Kari Steinmetz

(CC/T)

**University of Pennsylvania Scheie Eye Institute (Philadelphia, PA)**: Vatinee Bunya, MD

(PI); Michael Bezzerides (CC/T); Dominique Caggiano (CC/T); Sheri Drossner (T); Joan

Dupont (CC); Marybeth Keiser (CC/T); Mina Massaro, MD (CL); Stephen Orlin, MD (CL);

Ryan O’Sullivan (CC/T).

**Southern College of Optometry (Memphis, TN)**: Michael Christensen, OD PhD (PI);

Havilah Adkins (CC); Randy Brafford (CC/T); Cheryl Ervin (CL); Rachel Grant OD (CL);

Christina Newman (CL).

**Shettle Eye Research (Largo, FL)**: Lee Shettle, DO (PI); Debbie Shettle (CC).

**Stephen Cohen, OD, PC (Scottsdale, AZ)**: Stephen Cohen, OD (PI); Diane Rodman

(CC/T)

**Case Western Reserve University (Cleveland, OH)**: Loretta Szczotka-Flynn, OD PhD

(PI); Tracy Caster (T); Pankaj Gupta MD MS (CL); Sangeetha Raghupathy (CC/T); Rony

Sayegh, MD (CL).

**Mayo Clinic Arizona (Scottsdale, AZ)**: Joanne Shen, MD (PI); Nora Drutz, CCRC (CC);

Lauren Joyner, COA (T); Mary Mathis, COA (T); Michaele Menghini, CCRP (CC);

Charlene Robinson, CCRP (CC).

**Wolston & Goldberg Eye Associates (Torrance, CA)**: Damien Goldberg, MD (PI); Lydia

Jenkins (T); Brittney Rodriguez (CC/T); Jennifer Picone Jones (CC/T); Nicole Thompson

(T), Barry Wolstan, MD (CL).

**Northeast Ohio Eye Surgeons (Stow, OH)**: Marc Jones, MD (PI); April Lemaster (CC/T);

Julie Ransom-Chaney (T); William Rudy, OD (CL).

Tufts Medical Center (Boston, MA): Pedram Hamrah, MD (PI); Mildred Commodore

(CC); Christian Iyore (T); Lioubov Lazarev (T): Leah Mullen (T); Nicholas Pondelis (T);

Carly Satsuma (CC).

**University of Illinois at Chicago (Chicago, IL)**: Sandeep Jain, MD (PI); Peter Cowen

(CC/T); Joelle Hallak (CC); Christine Mun (CC/T); Roxana Toh (CC).

The Eye Centers of Racine & Kenosha (Racine, WI): Inder Singh, MD (PI); Pamela

Lightfield (CC/T); Eunice Lowery (T); Sarita Ornelas (T); R. Krishna Sanka, MD (CL);

Beth Saunders (T).

**Mulqueeny Eye Centers (St. Louis, MO)**: Sean P. Mulqueeny, OD (PI); Maggie Pohlmeier

(CC/T)

**Oculus Research at Garner Eyecare Center (Raleigh, NC)**: Carol Aune, OD (PI); Hoda

Gabriel (CC); Kim Major Walker, RN MS (CC/T); Jennifer Newsome (CC/T).

Resource Centers

**Chairman’s Office (Icahn School of Medicine at Mount Sinai, New York, NY)**: Penny

Asbell, MD, FACS, MBA (Study Chair); Brendan Barry (Clinical Research Coordinator);

Eric Kuklinski (Clinical Research Coordinator); Shir Levanon (Clinical Research Coordinator); Michael Farkouh, MD FRCPC, FACC, FAHA (Medical Safety Monitor);

Seunghee Kim-Schulze, PhD (Consultant); Robert Chapkin, PhD, MSc. (Consultant);

Giampaolo Greco, PhD (Consultant); Artemis Simopoulos, MD (Consultant); Ines Lashley

(Administrative Assistant); Peter Dentone, MD (Clinical Research Coordinator); Neha

Gadaria-Rathod, MD (Clinical Research Coordinator); Morgan Massingale, MS (Clinical Research Coordinator); Nataliya Antonova (Clinical Research Coordinator).

Coordinating Center (University of Pennsylvania Perelman School of Medicine

**Philadelphia, PA)**: Maureen G. Maguire, PhD (PI); Mary Brightwell-Arnold, SCP (Systems Analyst) John Farrar, MD PhD (Consultant); Sandra Harkins (Staff Assistant); Jiayan.

Huang, MS (Biostatistician); Kathy McWilliams, CCRP (Protocol Monitor); Ellen Peskin,

MA, CCRP (Director); Maxwell Pistilli, MS, MEd (Biostatistician); Susan Ryan (Financial

Administrator); Hilary Smolen (Research Fellow); Claressa Whearry (Administrative

Coordinator); Gui-Shuang Ying, PhD (Senior Biostatistician) Yinxi Yu (Biostatistician).

**Biomarker Laboratory (Icahn School of Medicine at Mount Sinai, New York, NY)**: Yi

Wei, PhD, DVM (co-Director, Biomarker Laboratory); Neeta Roy, PhD (co-Director,

Biomarker Laboratory); Seth Epstein, MD (Former co-Director; Biomarker Laboratory);

Penny A. Asbell, MD, FACS, MBA (Director and Study Chair).

Investigational Drug Service (University of Pennsylvania Perelman School of Medicine

**Philadelphia, PA)**: Kenneth Rockwell, Jr., PharmD MS (Director).

Peroxisomal Diseases Laboratory at the Kennedy Krieger Institute, Johns Hopkins

**University Baltimore MD**: Ann Moser (Co-Director/Consultant); Richard O. Jones, PhD.

(Co-Director/Consultant).

Meibomian Gland Reading Center (University of Pennsylvania Perelman School of

**Medicine, Philadelphia, PA)**: Ebenezer Daniel, MBBS, MPH, PhD, (PI); E. Revell Martin

(Image Grader); Candace Parker Ostroff, (Image Grader); Eli Smith (Image Grader); Pooja.

Axay Kadakia (Student Researcher).

National Eye Institute, National Institutes of Health, Department of Health and

**Human Services**: Maryann Redford, DDS, MPH (Program Officer).

Office of Dietary Supplements/National Institutes of Health, Department of Health and Human Services Committees

**Executive Committee (Members from all terms of appointment):** Penny Asbell, MD FACS,

MBA (Chair); Brendan Barry, MS; Munira Hussain, MS, COA, CCRP; Jack Greiner, MS,

OD, DO, PhD; Milton Hom, OD, FAAO; Holly Hindman, MD, MPH; Eric Kuklinski, BA;

Meng C. Lin OD, PhD. FAAO; Maureen G. Maguire, PhD; Kathy McWilliams, CCRP;

Ellen Peskin, MA, CCRP; Maryann Redford, DDS, MPH; Roni Shtein, MD, MS; Steven

Silverstein, MD; John Sutphin, MD.

**Operations Committee**: Penny Asbell, MD FACS, MBA (Chair); Brendan Barry, MS; Eric

Kuklinski, BA; Maureen G. Maguire, PhD; Kathleen McWilliams, CCRP, Ellen Peskin, MA,

CCRP; Maryann Redford, DDS, MPH.

**Clinic Monitoring Committee**: Ellen Peskin, MA, CCRP (Chair); Mary Brightwell Arnold, SCP, Maureen G. Maguire, PhD; Kathleen McWilliams, CCRP.

**Data and Safety Monitoring Committee**: Stephen Wisniewski, PhD (Chair); Tom Brenna,

PhD; William G. Christen Jr, SCD, OD, PhD; Jin-Feng Huang, PhD; Cynthia S. McCarthy,

DHCE, MA; Susan T. Mayne, PhD; Mari Palta, PhD; Oliver D. Schein, MD, MPH, MBA.

Industry Contributors of Products and Services

**Access Business Group, LLC (Ada, MI)** Jennifer Chuang, PhD. CCRP; Maydee Marchan,

M.Ch.E; Tian Hao, PhD; Christine Heisler; Charles Hu, PhD; Clint Throop, Vikas

Moolchandani, PhD.

Compounded Solutions in Pharmacy (Monroe, CT)

Leiter’s (San Jose, CA)

Immco Diagnostics Inc. (Buffalo NY

OCULUS Inc. (Arlington, WA)

RPS Diagnostics, Inc. (Sarasota, FL)

TearLab Corporation (San Diego, CA)

TearScience Inc. (Morrisville, NC).

## References

[1] Nichols KK, Foulks GN, Bron AJ, Glasgow BJ, Dogru M, Tsubota K, The international workshop on meibomian gland dysfunction: executive summary. Invest Ophthalmol Vis Sci 2011;52:1922–9. 21450913 10.1167/iovs.10-6997a PMC3072157

[2] Ajouz L, Nguyen A, Zhao C, Robinson MR, Nichols KK. Exploring signs and symptoms associated with meibomian gland dysfunction for use as clinical trial endpoints. J Ocul Pharmacol Ther 2023;39:611–21. 37643299 10.1089/jop.2023.0064 PMC10654652

[3] Stapleton F, Alves M, Bunya VY, Jalbert I, Lekhanont K, Malet F, TFOS DEWS II epidemiology report. Ocular Surf 2017;15:334–65. 10.1016/j.jtos.2017.05.003 28736337

[4] Viso E, Rodriguez-Ares MT, Abelenda D, Oubina B, Gude F. Prevalence of asymptomatic and symptomatic meibomian gland dysfunction in the general population of Spain. Invest Ophthalmol Vis Sci 2012;53:2601–6. 22427596 10.1167/iovs.11-9228

[5] Amano S, Inoue K. Estimation of the prevalence of meibomian gland dysfunction in Japan. Cornea 2017;36:684–8. 28410360 10.1097/ICO.0000000000001208

[6] Hassanzadeh S, Varmaghani M, Zarei-Ghanavati S, Heravian Shandiz J, Azimi KA. Global prevalence of meibomian gland dysfunction: a systematic review and meta-analysis. Ocul Immunol Inflamm 2021;29:66–75. 32589483 10.1080/09273948.2020.1755441

[7] Arita R, Minoura I, Morishige N, Shirakawa R, Fukuoka S, Asai K, Development of definitive and reliable grading scales for meibomian gland dysfunction. Am J Ophthalmol 2016;169:125–37. 27345733 10.1016/j.ajo.2016.06.025

[8] Bron AJ, Benjamin L, Snibson GR. Meibomian gland disease. Classification and grading of lid changes. Eye (Lond) 1991;5(Pt 4):395–411. 1743355 10.1038/eye.1991.65

[9] Foulks GN, Bron AJ. Meibomian gland dysfunction: a clinical scheme for description, diagnosis, classification, and grading. Ocul Surf 2003;1:107–26. 17075643 10.1016/s1542-0124(12)70139-8

[10] Pult H, Riede-Pult BH, Nichols JJ. Relation between upper and lower lids’ meibomian gland morphology, tear film, and dry eye. Optim Vis Sci 2012;89:310. 10.1097/OPX.0b013e318244e487 22246333

[11] Yamaguchi M, Kutsuna M, Uno T, Zheng XD, Kodama T, Ohashi Y. Marx line: fluorescein staining line on the inner lid as indicator of meibomian gland function. Am J Ophthalmol 2006;141:669–75. 16564801 10.1016/j.ajo.2005.11.004

[12] Deng YQ, Wang Q, Luo ZZ, Li SQ, Wang BW, Zhong J, Quantitative analysis of morphological and functional features in meibography for meibomian gland dysfunction: diagnosis and grading. Eclinicalmedicine 2021;40. 10.1016/j.eclinm.2021.101132 PMC8435692 34541482

[13] Asbell PA, Maguire MG, Peskin E, Bunya VY, Kuklinski EJ, Dry Eye A, Dry eye assessment and management (DREAM(c)) study: study design and baseline characteristics. Contemp Clin Trials 2018;71:70–9. 29883769 10.1016/j.cct.2018.06.002 PMC7250048

[14] Daniel E, Pistilli M, Ying GS, Bunya VY, Massaro-Giordano M, Asbell PA, Association of meibomian gland morphology with symptoms and signs of dry eye disease in the Dry Eye Assessment and Management (DREAM) study. Ocul Surf 2020;18:761–9. 32858234 10.1016/j.jtos.2020.07.014 PMC7686056

[15] Daniel E, Maguire MG, Pistilli M, Bunya VY, Massaro-Giordano GM, Smith E, Grading and baseline characteristics of meibomian glands in meibography images and their clinical associations in the Dry Eye Assessment and Management (DREAM) study. Ocul Surf 2019;17:491–501. 31022469 10.1016/j.jtos.2019.04.003 PMC6708483

[16] Pult H, Riede-Pult B. Comparison of subjective grading and objective assessment in meibography. Cont Lens Anterior Eye 2013;36:22–7. 23108007 10.1016/j.clae.2012.10.074

[17] Li JH, Ma JL, Hu M, Yu JQ, Zhao YN. Assessment of tear film lipid layer thickness in patients with Meibomian gland dysfunction at different ages. Bmc Ophthalmol 2020;20. 10.1186/s12886-020-01667-8 PMC7539430 33023522

[18] Nien CJ, Massei S, Lin G, Nabavi C, Tao J, Brown DJ, Effects of age and dysfunction on human meibomian glands. Arch Ophthalmol-Chic 2011;129:462–9. 10.1001/archophthalmol.2011.69 PMC4291168 21482872

[19] Moreno I, Verma S, Gesteira TF, Coulson-Thomas VJ. Recent advances in age-related meibomian gland dysfunction (ARMGD). Ocul Surf 2023;30:298–306. 37979775 10.1016/j.jtos.2023.11.003 PMC11092925

[20] Ruppenkamp J, Folly E, Holy C, Blackie CA. Clinical presentation, comorbidities and healthcare utilization of patients with dry eye and Meibomian gland dysfunction – a United States database analysis. Invest Ophthamol Vis Sci 2019;60.

[21] Finis D, Ackermann P, Pischel N, König C, Hayajneh J, Borrelli M, Evaluation of meibomian gland dysfunction and local distribution of meibomian gland atrophy by non-contact infrared meibography. Curr Eye Res 2015;40:982–9. 25330304 10.3109/02713683.2014.971929

[22] Srivastav S, Ali MH, Basu S, Singh S. Morphologic variants of Meibomian glands: age-wise distribution and differences between upper and lower eyelids. Front Med-Lausanne 2023;10. 10.3389/fmed.2023.1195568 PMC10507340 37731719

[23] Zhou NY, Edwards K, Colorado LH, Schmid KL. Lid margin score is the strongest predictor of Meibomian area loss. Cornea 2022;41:699–708. 35249979 10.1097/ICO.0000000000002913

[24] Tomlinson A, Bron AJ, Korb DR, Amano S, Paugh JR, Pearce EI, The international workshop on Meibomian gland dysfunction: report of the diagnosis subcommittee. Invest Ophthamol Vis Sci 2011;52:2006–49. 10.1167/iovs.10-6997f PMC3072162 21450918

[25] Schaumberg DA, Nichols JJ, Papas EB, Tong L, Uchino M, Nichols KK. The international workshop on Meibomian gland dysfunction: report of the subcommittee on the epidemiology of, and associated risk factors for, MGD. Invest Ophthamol Vis Sci 2011;52:1994–2005. 10.1167/iovs.10-6997e PMC3072161 21450917

[26] Arita R, Itoh K, Inoue K, Amano S. Noncontact infrared meibography to document age-related changes of the meibomian glands in a normal population. Ophthalmology 2008;115:911–5. 18452765 10.1016/j.ophtha.2007.06.031

[27] Den S, Shimizu K, Ikeda T, Tsubota K, Shimmura S, Shimazaki J. Association between meibomian gland changes and aging, sex, or tear function. Cornea 2006; 25:651–5. 17077655 10.1097/01.ico.0000227889.11500.6f

[28] Feng J, Wang J, Wu B, Shao Q, Zang Y, Cao K, Association of meibomian gland morphology with orifice plugging and lid margin thickening in meibomian gland dysfunction patients. Int Ophthalmol 2023;43:3207–18. 37140834 10.1007/s10792-023-02721-2

[29] Singh S, Naidu GC, Vemuganti G, Basu S. Morphological variants of meibomian glands: correlation of meibography features with histopathology findings. Brit J Ophthalmol 2023;107:195–200. 34417185 10.1136/bjophthalmol-2021-318876

[30] Singh S, Basu S. Morphological variants of Meibomian glands: correlation of meibography features with histopathology findings. Invest Ophthamol Vis Sci 2021:62. 10.1136/bjophthalmol-2021-318876 34417185

[31] Borchman D, Ramasubramanian A, Foulks GN. Human Meibum cholesteryl and wax ester variability with age, sex, and meibomian gland dysfunction. Invest Ophthalmol Vis Sci 2019;60:2286–93. 31112994 10.1167/iovs.19-26812 PMC6530518

[32] Sun MX, Moreno IY, Dang M, Coulson-Thomas VJ. Meibomian gland dysfunction: what have animal models taught us? Int J Mol Sci 2020;21. 10.3390/ijms21228822 PMC7700490 33233466

[33] Bu JH, Guo YL, Wu Y, Zhang RR, Zhuang JB, Zhao JK, Models for Meibomian gland dysfunction: in vivo and in vitro. Ocular Surf 2024;32:154–65. 10.1016/j.jtos.2024.03.003 38490475

[34] Yoon CH, Ryu JS, Hwang HS, Kim MK. Comparative analysis of age-related changes in lacrimal glands and meibomian glands of a C57BL/6 male mouse model. Int J Mol Sci 2020;21. 10.3390/ijms21114169 PMC7313015 32545199

[35] Huang BE, Fei FR, Wen H, Zhu Y, Wang ZZ, Zhang SW, Impacts of gender and age on meibomian gland in aged people using artificial intelligence. Front Cell Dev Biol 2023;11. 10.3389/fcell.2023.1199440 PMC10309028 37397262

[36] Craig JP, Wang MTM, Ambler A, Cheyne K, Wilson GA. Characterising the ocular surface and tear film in a population-based birth cohort of 45-year old New Zealand men and women. Ocular Surf 2020;18:808–13. 10.1016/j.jtos.2020.08.005 32798736

[37] Wang JY, Li SX, Yeh TN, Chakraborty R, Graham AD, Yu SX, Quantifying Meibomian gland morphology using artificial intelligence. Optometry Vis Sci 2021; 98:1094–103. 10.1097/OPX.0000000000001767 PMC8484036 34469930

[38] Wang JY, Yeh TN, Chakraborty R, Yu SX, Lin MC. A deep learning approach for Meibomian gland atrophy evaluation in meibography images. Transl Vis Sci Technol 2019;8. 10.1167/tvst.8.6.37 PMC6922272 31867138

[39] Teo CHY, Ong HS, Liu YC, Tong L. Meibomian gland dysfunction is the primary determinant of dry eye symptoms: analysis of 2346 patients. Ocular Surf 2020;18: 604–12. 10.1016/j.jtos.2020.06.008 32682082

[40] Wu XD, Chen X, Ma YJ, Lin XQ, Yu XW, He SH, Analysis of tear inflammatory molecules and clinical correlations in evaporative dry eye disease caused by meibomian gland dysfunction. Int Ophthalmol 2020;40:3049–58. 32601963 10.1007/s10792-020-01489-z PMC7550292

[41] Wang JY, Graham AD, Yu SX, Lin MC. Predicting demographics from meibography using deep learning. Sci Rep-UK 2022;12. 10.1038/s41598-022-18933-y PMC9489726 36127431

[42] Schaumberg DA, Sullivan DA, Buring JE, Dana MR. Prevalence of dry eye syndrome among US women. Am J Ophthalmol 2003;136:318–26. 12888056 10.1016/s0002-9394(03)00218-6

[43] Watson SL, Jones LW, Stapleton F, Hinds M, Ng A, Tan J, Efficacy and safety of AZR-MD-001 selenium sulfide ophthalmic ointment in adults with meibomian gland dysfunction: a vehicle-controlled, randomized clinical trial. Ocular Surf 2023;29:537–46. 10.1016/j.jtos.2023.07.002 37478969

[44] Lienert JP, Tarko L, Uchino M, Christen WG, Schaumberg DA. Long-term natural history of dry eye disease from the patient’s perspective. Ophthalmology 2016;123: 425–33. 26610720 10.1016/j.ophtha.2015.10.011 PMC4724500

